# The Impact of Dual Antiplatelet Therapy Guided by Platelet Function Testing on the Prognosis of Patients With Dual High-Risk Acute Coronary Syndrome Undergoing Percutaneous Coronary Intervention

**DOI:** 10.31083/RCM41544

**Published:** 2026-02-11

**Authors:** Mengjie Lei, Yachao Li, Jingyao Wang, Xue Sun, Cairong Li, Xiao Wang, Zhigang Zhao, Zengming Xue

**Affiliations:** ^1^Langfang Core Laboratory of Precision Treatment of coronary artery disease, Langfang People's Hospital, 065000 Langfang, Hebei, China

**Keywords:** dual antiplatelet therapy, platelet function testing, dual high-risk, net clinical adverse events, acute coronary syndrome

## Abstract

**Background:**

To investigate the effect of dual antiplatelet therapy (DAPT) guided by platelet function testing (PFT) on the prognosis of patients with acute coronary syndrome (ACS) at a high risk for ischemia and bleeding who underwent percutaneous coronary intervention (PCI).

**Methods:**

A retrospective analysis was conducted on 1816 patients with ACS and a dual high risk who underwent PCI at a single center from March 2017 to November 2022. Patients were stratified into the guided DAPT group (n = 712) and standard DAPT group (n = 1104) according to whether the patient received PFT. All patients received oral DAPT for a duration of 12 months post-PCI. The deadline for the endpoint was within 12 months of receiving PCI. The primary endpoint was the number of net clinical adverse events (NACEs) that occurred during follow-up, including the composite endpoint of major adverse cardiovascular and cerebrovascular events (MACCEs) and bleeding, as defined by the bleeding academic research consortium (BARC) (type 3 or greater).

**Results:**

Compared with the standard DAPT group, the guided DAPT group exhibited a lower incidence of NACEs (4.8% vs. 8.7%; *p *= 0.001), MACCEs (3.9% vs. 6.7%; *p *= 0.017), cardiac death (0.4% vs*.* 1.5%; *p *= 0.038), and stroke (0.6% vs. 2.5%; *p *= 0.005) during follow-up. Cox regression analysis revealed that the incidence of NACEs (hazard ratio (HR): 0.543, 95% confidence interval (CI): 0.363–0.812; *p *= 0.003), MACCEs (HR: 0.570, 95% CI: 0.364–0.893; *p *= 0.014), cardiac death (HR: 0.249, 95% CI: 0.072–0.866; *p* = 0.029), and stroke (HR: 0.174, 95% CI: 0.060–0.501; *p* = 0.001) in the guided DAPT group was 0.543, 0.570, 0.249, and 0.174 times, respectively, that in the standard DAPT group.

**Conclusion:**

For patients with ACS who are at high risk in the East Asian population, the primary recommendation is to use PFT to guide DAPT within 12 months after PCI, which can reduce the incidence of NACEs, primarily by lowering the rate of MACCEs.

## 1. Introduction

According to current guidelines, acute coronary syndrome (ACS) patients undergoing percutaneous coronary intervention (PCI) should receive dual antiplatelet therapy (DAPT) for a minimum of 12 months, with a preference for potent P2Y_12_ receptor inhibitors [1, 2, 3]. These agents are key for thrombosis prevention and reducing cardiovascular risk. However, the protection that DAPT provides against thrombotic events comes at the cost of an elevated bleeding risk. Therefore, risk stratification and personalized DAPT strategies for patients undergoing PCI will help maximize net clinical benefit [4]. With the continuous development of precision medicine, related research has increasingly focused on methods for assessing ischemic and bleeding risks in ACS patients [4, 5, 6, 7] to help the identify ACS patients with high-risk features as well as to formulate precise DAPT strategies. In order to stratify the high risk, multiple guidelines and study have defined high-risk populations for ischemia or bleeding: the definition of high-risk in the 2023 European Society of Cardiology (ESC) guidelines, the Academic Research Consortium for High Bleeding Risk (ARC-HBR) criteria, and the derivation and validation of the predicting bleeding complications in patients undergoing stent implantation and subsequent dual antiplatelet therapy (PRECISE-DAPT) score, etc. [5]. Potent P2Y_12_ receptor inhibitors or extended duration of DAPT may be a therapeutic consideration for patients with high risk ischemic features, whereas clopidogrel or shortened DAPT duration may be appropriate for patients with high bleeding risk. However, for patients with dual high-risks, determining the optimal DAPT strategy based on risk assessment alone is not straightforward because there is a greater need to balance ischemic and bleeding risk in these patients. Currently, the optimal DAPT strategy within 12 months after PCI for dual high-risk ACS patients has not been firmly established.

Despite advancements in defining antiplatelet strategies for the chronic phase beyond one year post-PCI, a critical evidence gap remains regarding the optimal antiplatelet regimen during the first year following PCI, specifically in patients at dual high-risk. While clinical trials, such as the OPT-BIRISK (optimal antiplatelet therapy for high bleeding and ischemic risk) trial has focused on determining the strategy after the standard DAPT period [5], there is a distinct lack of randomized or large-scale data guiding the initial strategy within this first year. This gap is particularly relevant for dual high-risk patients, in whom the balance between preventing thrombotic events and mitigating bleeding complications is most delicate.

Platelet function testing (PFT) can individualize the responses to platelet activation and aggregation capacity, ensuring that patients with ACS receive adequate platelet inhibition during long-term treatment. Recognizing that PFT serves as a predictor for ischemic and bleeding events in post-PCI patients, researchers have used PFT to adjust antiplatelet therapy. The 2020 ESC guidelines for non-ST-segment elevation ACS (NSTE-ACS) state that de-escalation is presented as a viable alternative in patients with ACS who are unsuitable for intensive therapy. This de-escalation may be based on clinical judgment or guided by PFT or genetic testing, depending on the risk profiles of patients and the feasibility of testing, although it is only a class IIb recommendation [3]. For patients requiring DAPT escalation, platelet function may be oversuppressed, thereby requiring adjustment under guidance. The platelet function monitoring to adjust antiplatelet therapy in elderly patients stented for an acute coronary syndrome (ANTARCTIC) trial, which adopted a mixed strategy of adjusting therapy (either escalation or de-escalation) based on PFT results in 877 ACS patients aged 75 and above after PCI. However, this trial failed to demonstrate that the PFT-guided strategy significantly reduced the net adverse clinical events [8]. This negative outcome suggests that the utility of a PFT-guided strategy remains uncertain. Consequently, there is currently no clear consensus regarding the precise implementation of PFT in clinical care. Key questions include whether it holds greater value in guiding de-escalation rather than escalation, or in selected high-risk subgroups. A meta-analysis showed that PFT-guided medication adjustment, compared to empirical medication adjustment, could reduce the risks of cardiac death, recurrent myocardial infarction, in-stent thrombosis, stroke, and minor bleeding; however, no differences in all-cause mortality and major bleeding were found [9]. The current studies have not identified the optimal population for PFT. Therefore, the reason for this study which was the investigation and outcomes of PFT in dual high risk patients because even in other populations with different risk profiles prove its efficacy and safety.

## 2. Materials and Methods

### 2.1 Study Participants

This single-centre retrospective cohort study included patients with dual high-risk ACS who underwent PCI at the Cardiology Department of Langfang People’s Hospital from March 2017 to November 2022. All patients with ACS included in this study met the relevant diagnostic criteria outlined in the ESC guidelines [1, 3]. Clinical Presentation: acute chest discomfort (or chest pain-equivalent signs/symptoms)-pain, pressure, tightness, heaviness, or burning. Electrocardiogram (ECG): STEMI (persistent ST-segment elevation (or ST-segment elevation equivalents) on ECG); NSTE-ACS (transient ST-segment elevation, persistent or transient ST-segment depression, and T wave abnormalities, including hyperacute T waves, T wave inversion, biphasic T waves, flat T waves, and pseudo-normalization of T waves. Alternatively, the ECG may be normal). Biomarkers: STEMI/non-ST-elevation myocardial infarction (NSTEMI) -a significant rise and/or fall of cTn, with at least one value above the 99th percentile upper reference limit. The patients met the OPT-BIRISK trial-defined criteria for the dual risk of ischemia and bleeding (Table 1) [7]. The study population was categorized into two groups according to whether they received PFT-guided DAPT: the guided DAPT group and standard DAPT group.

**Table 1.** **High risk criteria**.

Dual high risk	High ischaemia risk	High bleeding risk
(at least one of the criteria must be met)	(at least one of the criteria must be met)	(at least one of the criteria must be met)
(1) age ≥75 years;	(1) multivessel coronary disease;	(1) female sex;
(2) age of 65–74 years with high ischemia or high bleeding risks;	(2) total stent length >30 mm;	(2) iron deficiency anaemia;
(3) age <65 years with high ischemia and high bleeding risks.	(3) presence of thrombotic lesions;	(3) history of haemorrhagic or ischemic stroke;
	(4) bifurcation lesions requiring double stent treatment (Medina classification 0, 1, 1 or 1, 1, 1);	(4) diabetes requiring medication (oral hypoglycaemic therapy or subcutaneous insulin injection);
	(5) lesions in the left anterior descending artery (≥50%) or proximal left anterior descending artery (≥70%);	(5) chronic kidney disease, defined as estimated glomerular filtration rate <60 mL/min/1.73 m^2^ or creatinine clearance rate <60 mL/min.
	(6) calcified lesions requiring rotational atherectomy;	
	(7) acute coronary syndrome with positive troponin levels;	
	(8) diagnosis of vascular disease, including previous myocardial infarction, ischemic stroke, peripheral artery disease (PAD), or coronary atherosclerotic heart disease (CAD)/PAD vascular reconstruction;	
	(9) recurrent myocardial infarction, coronary revascularization, stent thrombosis, or stroke occurring within 9 months prior to percutaneous coronary intervention;	
	(10) diabetes requiring medication (oral hypoglycaemic therapy or subcutaneous insulin injection);	
	(11) chronic kidney disease, defined as estimated glomerular filtration rate <60 mL/min/1.73 m^2^ or creatinine clearance rate <60 mL/min.	

The inclusion criteria were as follows: (1) age ≥18 years; (2) follow-up duration ≥12 months; (3) all PCI patients who exhibited typical symptoms of myocardial ischaemia or myocardial infarction, electrocardiogram changes, or relevant laboratory examination results; and (4) all patients with ACS who were deemed to have a dual high risk based on the OPT-BIRISK score. The exclusion criteria were as follows: (1) patients allergic to aspirin or any P2Y_12_ receptor inhibitor, or those who experienced severe adverse reactions that could prevent continued medication (e.g., significant bradycardia, intolerable dyspnea); (2) patients who discontinued medication for any reason or failed to adhere to DAPT for 12 months; (3) patients with diseases that could severely affect the platelet count and function (e.g., severe rheumatic or immune diseases, aplastic anaemia); (4) patients with severe hepatic or renal insufficiency (Child-Pugh class 2/3 or estimated glomerular filtration rate [eGFR] <30 mL/min/1.73 m^2^); and (5) patients participating in other research projects related to antiplatelet and anticoagulant therapy during the follow-up period.

### 2.2 Baseline Data Collection

The case report form (CRF) includes patient baseline and prognostic data. These data included general information, diagnosis, and treatment of patients during hospitalization, including age, sex, body mass index (BMI), primary PCI, history of chronic diseases, laboratory examination, culprit artery, and PCI details. Baseline patient data were collected from electronic medical records, while the grouping and prognostic data for CRF were acquired via telephone follow-ups and outpatient visits. For patients in the guided DAPT group, PFT and medication adjustments at 3 months after PCI were completed during outpatient visits. After completing the CRF for each patient, the relevant data were entered into SPSS software version 26.0 (IBM Corp., Armonk, NY, USA) by two individuals to ensure accuracy of the data. The follow-up period was 12 months after PCI.

### 2.3 Definitions

#### 2.3.1 Definition of the Study Protocol

In the guided DAPT group, fasting venous blood was obtained from the elbow at 3 months after PCI. 3 mL of venous blood was collected into a coagulation vacuum tube, thoroughly mixed for anticoagulation, and tested within 3 hours at room temperature. Blood was placed in a tube containing sodium citrate anticoagulant (20231208A, Shenzhen Kangnaige Biological Technology Co., Ltd., Shenzhen, Guangdong, China) and centrifuged at 150 ×g for 10 minutes. The upper serum layer was then transferred to an EP tube and centrifuged again at 2000 ×g for 10 minutes. Based on continuous-counting multi-parameter platelet function analyzer (AG80 fully automatic platelet aggregometer, Shandong Tell Medical Technology Co., Ltd., Jinan, Guangdong, China), the light transmission aggregometry (LTA) was used to measure the maximum aggregation rate (MAR) of adenosine diphosphate (ADP), referred to as MAR(ADP). A 250 µL aliquot of the well-mixed sample was transferred into a reaction tube cup. ADP reagents (Shandong Telixin Medical Technology Co., Ltd.,Weihai, Shandong, China) were added to the test sample to induce platelet aggregation, followed by immediate analysis. Platelet counts in the blood sample were measured at fixed intervals over time. Upon obtaining the lowest platelet count, the result was automatically converted to evaluate the MAR(ADP) (**Supplementary Material 1**).

At 3 months after PCI, the DAPT regimen for patients in the guided DAPT group was adjusted based on PFT results as follows (decision tree: **Supplementary Material 2**):

(1) For patients whose DAPT regimen within 3 months after PCI was aspirin (100 mg quaque die (QD)) + clopidogrel (75 mg QD): if MAR(ADP) was ≤50%, the original regimen was maintained; if MAR(ADP) was >50%, the DAPT regimen was changed to aspirin (100 mg QD) + ticagrelor (60 mg/90 mg bis in die (BID)), with the dose of ticagrelor determined based on post-administration MAR(ADP).

(2) For patients whose DAPT regimen within 3 months after PCI was aspirin (100 mg QD) + ticagrelor (60 mg BID): if MAR(ADP) was ≤30%, the DAPT regimen was changed to aspirin (100 mg QD) + clopidogrel (75 mg QD), and MAR(ADP) was rechecked after 1 week of medication; if 30% ≤ MAR(ADP) ≤ 50%, the original regimen was maintained; if MAR(ADP) was >50%, the DAPT regimen was changed to aspirin (100 mg QD) + ticagrelor (90 mg BID).

(3) For patients whose DAPT regimen within 3 months after PCI was aspirin (100 mg QD) + ticagrelor (90 mg BID): if MAR(ADP) was ≤30%, the DAPT regimen was changed to aspirin (100 mg QD) + clopidogrel (75 mg QD)/ticagrelor (60 mg BID), with the choice between two P2Y_12_ receptor inhibitors determined based on post-administration MAR(ADP); if MAR(ADP) was >30%, the original regimen was maintained.

For patients in the standard DAPT group, the DAPT regimen within 3 months after PCI was aspirin (100 mg QD), clopidogrel (75 mg QD), ticagrelor (60 mg BID), and ticagrelor (90 mg BID). At 3 months, adjustments to the DAPT regimen were made based on the clinical judgment of the physician.

A minimum 12-month duration of DAPT was implemented in all patients.

#### 2.3.2 Definition of the Outcome Indicators

In this study, the primary endpoint was the net clinical adverse events (NACE) during the follow-up period, which included a composite endpoint of major adverse cardiovascular and cerebrovascular events (MACCE) and bleeding academic research consortium (BARC) bleeding (type 3 or greater). The secondary endpoint was MACCE, which included a composite endpoint of cardiac death, myocardial infarction, ischemia-driven revascularization, and stroke. The safety endpoint was BARC bleeding, including BARC bleeding (type 3 or greater) and BARC bleeding (type 1 or 2).

### 2.4 Follow-up

The department trained data clerks to follow up on all patients. Visits were scheduled at 2 weeks, 3 months, 6 months, and 1 year after PCI. For patients who did not attend outpatient follow-ups, telephone follow-ups were conducted to track the endpoints. For patients in the guided DAPT group, if adjustments to the DAPT regimen were made at 3 months after PCI, outpatient follow-ups were still required when PFT was performed. The endpoint deadline was within 12 months after PCI.

### 2.5 Statistical Analysis

Statistical analysis was performed using IBM SPSS Statistics (version 26.0). Normally distributed continuous data were expressed as mean ± standard deviation, and intergroup comparisons were performed using the *t*-test. Non-normally distributed continuous data were presented as medians with interquartile ranges, and intergroup comparisons were performed using the rank-sum test. Categorical data were described as frequencies and percentages, and intergroup comparisons were conducted using the chi-square test. Multivariate Cox regression analysis was used to correct for confounding factors, including variables with statistically significant differences in the baseline data and those that might significantly affect the outcome indicators. The Kaplan–Meier survival curve was used to analyse the survival rates of the two groups. A two-tailed test was applied, with *p* ≤ 0.05 being considered statistically significant. For patients lost to follow-up, a sensitivity analysis was conducted by imputing data under two scenarios: assuming that these patients experienced endpoints and assuming they did not. The NACE analysis was repeated on these two hypothetical datasets to verify the stability of the outcomes.

## 3. Results

### 3.1 Participant Screening Process 

**Fig. 1.**
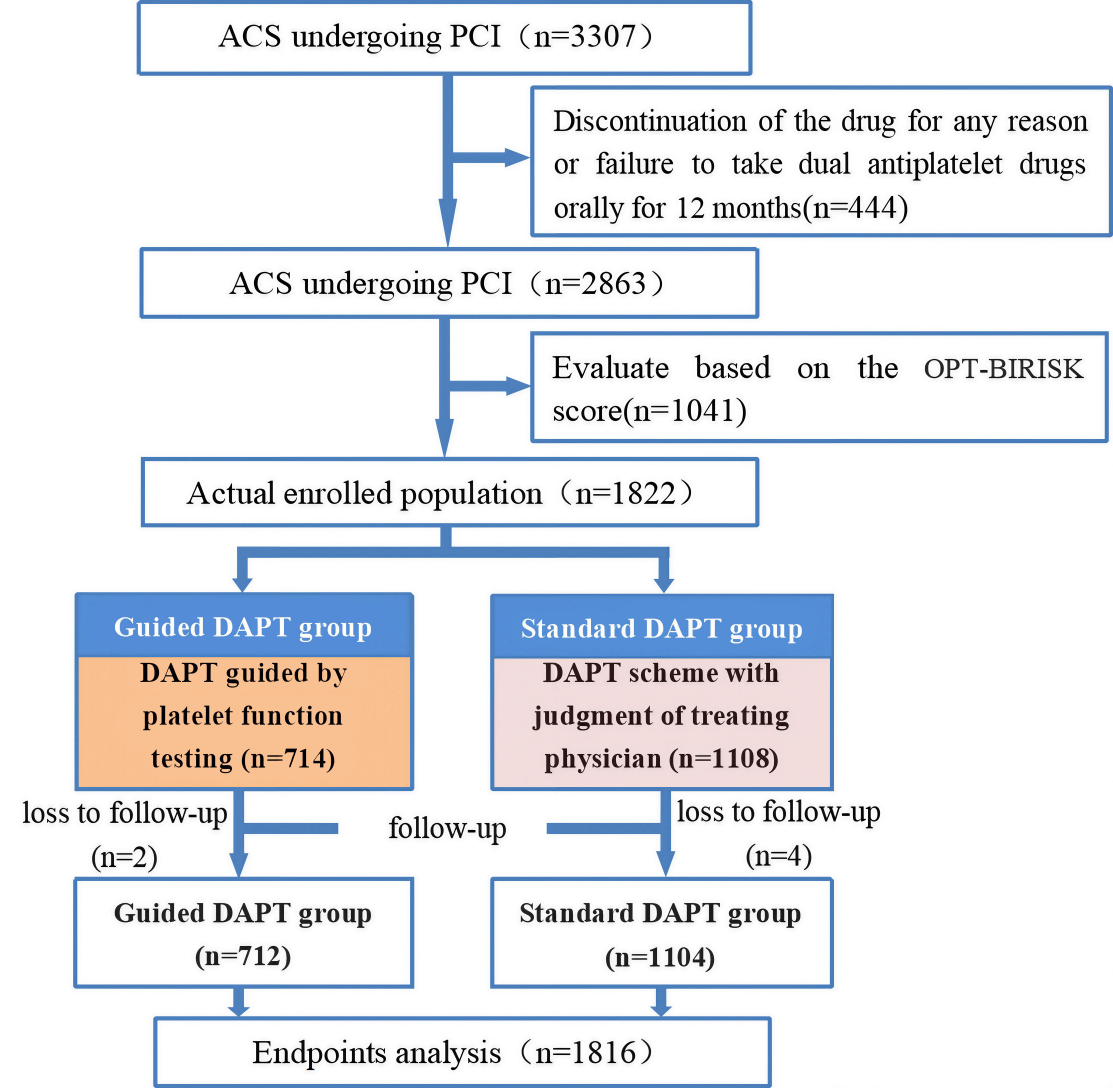
**Flowchart**. OPT-BIRISK, optimal antiplatelett herapy for high bleeding and ischemic RISK patients; ACS, acute coronary syndrome; PCI, percutaneous coronary intervention; DAPT, dual antiplatelet therapy.

This study screened participants among 3307 ACS patients who underwent PCI. After applying the inclusion and exclusion criteria, 1822 dual high-risk patients were enrolled. During the 12-month follow-up period, two patients in the guided DAPT group were lost to follow-up, resulting in the final inclusion of 712 patients. In the standard DAPT group, four patients were lost to follow-up, leading to the final inclusion of 1104 patients. The mean follow-up duration in this study was 40.70 ± 21.45 months. The endpoint deadline was within 12 months after PCI (Fig. 1).

### 3.2 Baseline Characteristics

No statistically significant differences between the two groups were detected in terms of age, female sex, BMI, presentation, hypertension, type 2 diabetes, cerebrovascular disease, previous myocardial infarction, atrial fibrillation, smoking, family history of coronary atherosclerotic heart disease (CAD), OPT-BIRISK, optimal antiplatelet antiplatelet therapy for Chinese patients with coronary artery disease (OPT-CAD), ARC-HBR and ticagrelor at discharge (all *p* > 0.05). There is a difference in the proportion of presentations and the types of P2Y_12_ inhibitors 3–12 months after PCI between the two groups (all *p* < 0.001). The proportion of primary PCI was significantly lower in the guided DAPT group than in the standard DAPT group (*p* < 0.001). In the PFT-guided group, the proportion of patients who switched P2Y_12_ inhibitors was 30.6% (218/712) (Table 2).

**Table 2.** **Baseline characteristics**.

Characteristic		Guided DAPT group (n = 712)	Standard DAPT group (n = 1104)	*t*/χ^2^ value	*p* value
Age (years; m ± SD)		63.51 ± 9.71	63.80 ± 9.15	0.638	0.524
Female (n, %)		331 (46.5%)	468 (42.4%)	2.949	0.086
BMI (kg/m^2^; m ± SD)		25.76 ± 3.07	25.86 ± 2.65	0.723	0.470
Presentation (n, %)					
	UAP	411 (57.7%)	512 (46.4%)	29.139	<0.001^ab^
	NSTEMI	50 (7.0%)	61 (5.5%)		
	STEMI	251 (35.3%)	531 (48.1%)		
Primary PCI (n, %)		180 (25.3%)	457 (41.4%)	49.354	<0.001
Medical history (n, %)					
	Hypertension	527 (74.0%)	782 (70.8%)	2.180	0.140
	Type 2 diabetes	250 (35.1%)	362 (32.8%)	1.045	0.307
	Cerebrovascular disease	125 (17.6%)	187 (16.9%)	0.116	0.733
	OMI	56 (7.9%)	70 (6.3%)	1.558	0.212
	Atrial fibrillation	16 (2.2%)	33 (3.0%)	0.908	0.341
	Current smoker	298 (41.9%)	451 (40.9%)	0.180	0.672
	CAD family history	30 (4.2%)	48 (4.3%)	0.019	0.890
	High ischaemia risk of OPT-BIRISK (n, %)	698 (98.0%)	1093 (99.0%)	2.999	0.083
OPT-CAD (n, %)					
	Low risk	227 (31.9%)	389 (35.2%)	2.805	0.246
	Medium risk	430 (60.4%)	623 (56.4%)		
	High risk	55 (7.7%)	92 (8.3%)		
ARC-HBR (n, %)		186 (26.1%)	278 (25.2%)	0.202	0.653
Ticagrelor at discharge (n, %)		327 (45.9%)	541 (49.0%)	1.642	0.200
Switch of P2Y_12_ inhibitors		218 (30.6%)	-	-	-
Types of P2Y_12_ inhibitors 3–12 months after PCI (n, %)					
	Clopidogrel 75 mg	446 (62.6%)	692 (62.7%)	17.158	<0.001
	Ticagrelor 90 mg	224 (31.5%)	387 (35.1%)		
	Ticagrelor 60 mg	42 (5.9%)	25 (2.3%)		

M, mean; SD, standard deviation; BMI, body mass index; UAP, unstable angina pectoris (a. *p* < 0.001); NSTEMI, non-ST segment elevation myocardial infarction; STEMI, ST segment elevation myocardial infarction (b. *p* < 0.001); OMI, old myocardial infarction; CAD, coronary atherosclerotic artery disease; OPT-BIRISK, optimal antiplatelet therapy for high bleeding and ischemic risk; OPT-CAD, optimal antiplatelet antiplatelet therapy for Chinese patients with coronary artery disease; ARC-HBR, the academic research consortium for high bleeding risk; Types of P2Y_12_ inhibitors 3–12 months after PCI: clopidogrel 75 mg vs. ticagrelor 60 mg (*p* < 0.001); ticagrelor 90 mg *vs.* ticagrelor 60 mg (*p* < 0.001).

### 3.3 Laboratory Parameters

There were no statistically significant differences in baseline laboratory parameters, including white blood cell count, hemoglobin, platelet count, fibrinogen, eGFR, uric acid, fasting blood glucose, and triglycerides, between the two groups (all *p* > 0.05). The total cholesterol levels were significantly higher in the guided DAPT group than in the standard DAPT group (*p* < 0.001). The low-density lipoprotein levels were significantly higher in the guided DAPT group than in the standard DAPT group (*p* = 0.002). The high-density lipoprotein levels were significantly higher in the guided DAPT group than in the standard DAPT group (*p* = 0.004) (Table 3).

**Table 3.** **Laboratory tests**.

Characteristic	Guided DAPT group (n = 712)	Standard DAPT group (n = 1104)	*t* value	*p* value
WBC (×10^9^/L)	7.40 ± 2.35	7.54 ± 2.39	1.230	0.219
HGB (g/L)	134.73 ± 15.54	134.04 ± 14.94	0.936	0.350
PLT (×10^9^/L)	234.30 ± 56.81	233.29 ± 55.27	0.375	0.707
FIB (g/L)	3.44 ± 0.84	3.44 ± 0.90	0.081	0.935
eGFR	92.72 ± 16.40	92.14 ± 16.21	0.739	0.460
UA (µmol/L)	320.75 ± 90.81	317.46 ± 83.39	0.780	0.435
FBG (mmol/L)	6.95 ± 2.51	7.12 ± 2.43	1.467	0.142
TC (mmol/L)	4.92 ± 1.42	4.67 ± 1.14	3.089	<0.001
TG (mmol/L)	1.95 ± 1.21	1.88 ± 1.00	1.345	0.179
LDL-C (mmol/L)	2.93 ± 0.93	2.80 ± 0.80	3.065	0.002
HDL-C (mmol/L)	1.13 ± 0.34	1.09 ± 0.27	2.882	0.004

WBC, white blood cell; HGB, hemoglobin; PLT platelet; FIB, fibrinogen; eGFR, estimated glomerular filtration rate; UA, uric acid; FBG, fasting blood glucose; TC, total cholesterol; TG, triglyceride; LDL-C, low density lipoprotein-cholesterol; HDL-C, high density lipoprotein-cholesterol.

### 3.4 Coronary Angiography and PCI

No statistically significant differences between the two groups were detected in terms of the culprit artery, chronic total occlusion, in-stent restenosis, proximal segment of the left anterior descending artery, and number of vessels with lesions (all *p* > 0.05). The proportion of patients with ostial lesions was significantly higher in the guided DAPT group than in the standard DAPT group (*p* < 0.009). The proportion of patients with diffuse lesions was significantly higher in the guided DAPT group than in the standard DAPT group (*p* = 0.005). The proportion of patients with small-vessel disease was significantly lower in the guided DAPT group than in the standard DAPT group (*p* < 0.001). The proportion of patients achieving complete revascularization was significantly lower in the guided DAPT group than in the standard DAPT group (*p* < 0.001). The number of stents used was significantly higher in the guided DAPT group than in the standard DAPT group (*p* = 0.002) (Table 4).

**Table 4.** **Procedural characteristics**.

Characteristic		Guided DAPT group (n = 712)	Standard DAPT group (n = 1104)	*t/χ^2^* value	*p* value
Culprit artery (n, %)					
	LM	15 (2.1%)	33 (3.0%)	1.310	0.252
	LAD	637 (89.5%)	994 (90.0%)	0.154	0.695
	LCX	506 (71.1%)	780 (70.7%)	0.036	0.849
	RCA	555 (77.9%)	856 (77.5%)	0.043	0.836
Ostial lesion (n, %)		231 (32.4%)	295 (26.7%)	6.890	0.009
Diffused lesion (n, %)		378 (53.1%)	512 (46.4%)	7.806	0.005
CTO (n, %)		7 (1.0%)	24 (2.2%)	3.658	0.056
ISR (n, %)		19 (2.7%)	18 (1.6%)	2.337	0.126
Small vessel (n, %)		52 (7.3%)	178 (16.1%)	30.441	<0.001
LADp (n, %)		133 (18.7%)	190 (17.2%)	0.639	0.424
Complete RV (n, %)		168 (23.6%)	398 (36.1%)	31.30	<0.001
Number of lesion vessel (n; m ± SD)		2.42 ± 0.74	2.43 ± 0.73	0.434	0.664
Number of stents (n; m ± SD)		1.46 ± 0.65	1.37 ± 0.62	3.053	0.002

LM, left main; LAD, left anterior descending; LCX, left circumflex coronary artery; RCA, right coronary artery; CTO, chronic total occlusion; ISR, In-stent restenosis; LADp, proximal segment of left anterior descending; RV, revascularization.

### 3.5 Endpoints During Follow-up

The results showed that, compared to the standard DAPT group, the guided DAPT group had significantly lower incidence of NACE, BARC bleeding (type 3 or greater), cardiac death, and stroke within 0–12 months after PCI (*p* = 0.018, *p* = 0.029, *p* = 0.038, and *p* = 0.007, respectively). During the follow-up period, there were no statistically significant differences in the incidence of MACCE, myocardial infarction, target vessel revascularization, BARC bleeding, or BARC bleeding (type 1 or 2) between the two groups (all *p* > 0.05) (Table 5).

**Table 5.** **Endpoints during follow-up**.

Characteristic		Guided DAPT group (n = 712)	Standard DAPT group (n = 1104)	*HR*	*95% CI*	*p*
Endpoints within 0–12 months						
	NACE (n, %)	43 (6.0%)	102 (9.3%)	0.651	0.456–0.930	0.018
	MACCE (n, %)	38 (5.3%)	79 (7.2%)	0.766	0.520–1.129	0.178
	Cardiac death (n, %)	3 (0.4%)	17 (1.5%)	0.273	0.080–0.932	0.038
	MI (n, %)	16 (2.3%)	24 (2.2%)	1.033	0.549–1.944	0.921
	TVR (n, %)	23 (3.3%)	25 (2.3%)	1.427	0.810–2.515	0.218
	Stroke (n, %)	5 (0.7%)	29 (2.7%)	0.269	0.104–0.694	0.007
	BARC bleeding events (n, %)	131 (18.4%)	183 (16.6%)	1.116	0.892–1.397	0.337
	Type 3 or greater	6 (0.8%)	25 (2.3%)	0.370	0.152–0.902	0.029
	Type 1 or 2	126 (17.7%)	167 (15.1%)	1.179	0.936–1.486	0.163
Endpoints within 3–12 months						
	NACE (n, %)	34 (4.8%)	96 (8.7%)	0.525	0.353–0.779	0.001
	MACCE (n, %)	28 (3.9%)	74 (6.7%)	0.588	0.380–0.908	0.017
	Cardiac death (n, %)	3 (0.4%)	17 (1.5%)	0.273	0.080–0.932	0.038
	MI (n, %)	11 (1.5%)	22 (2.0%)	0.798	0.387–1.648	0.543
	TVR (n, %)	19 (2.7%)	22 (2.0%)	1.407	0.760–2.605	0.277
	Stroke (n, %)	4 (0.6%)	28 (2.5%)	0.224	0.079–0.638	0.005
	BARC bleeding events (n, %)	83 (11.7%)	105 (9.5%)	1.239	0.929–1.652	0.145
	Type 3 or greater	6 (0.8%)	22 (1.9%)	0.418	0.170–1.032	0.059
	Type 1 or 2	77 (10.8%)	90 (8.2%)	1.347	0.994–1.826	0.055

NACE, net adverse clinical event; MACCE, major adverse cardiovascular and cerebrovascular event; MI, myocardial infarction; TVR, target vessel revascularization; BARC, bleeding academic research consortium.

The results showed that, compared to the standard DAPT group, the guided DAPT group had significantly lower incidence of NACE, MACCE, cardiac death, and stroke within 3–12 months after PCI (*p* = 0.001, *p* = 0.017, *p* = 0.038 and *p* = 0.005, respectively). There were no statistically significant differences in the incidence of myocardial infarction, target vessel revascularization, BARC bleeding, BARC bleeding (type 3 or greater), or BARC bleeding (type 1 or 2) within 3–12 months after PCI between the two groups (*p* = 0.077) (Table 5).

### 3.6 Kaplan‒Meier Survival Curve Analysis

**Fig. 2.**
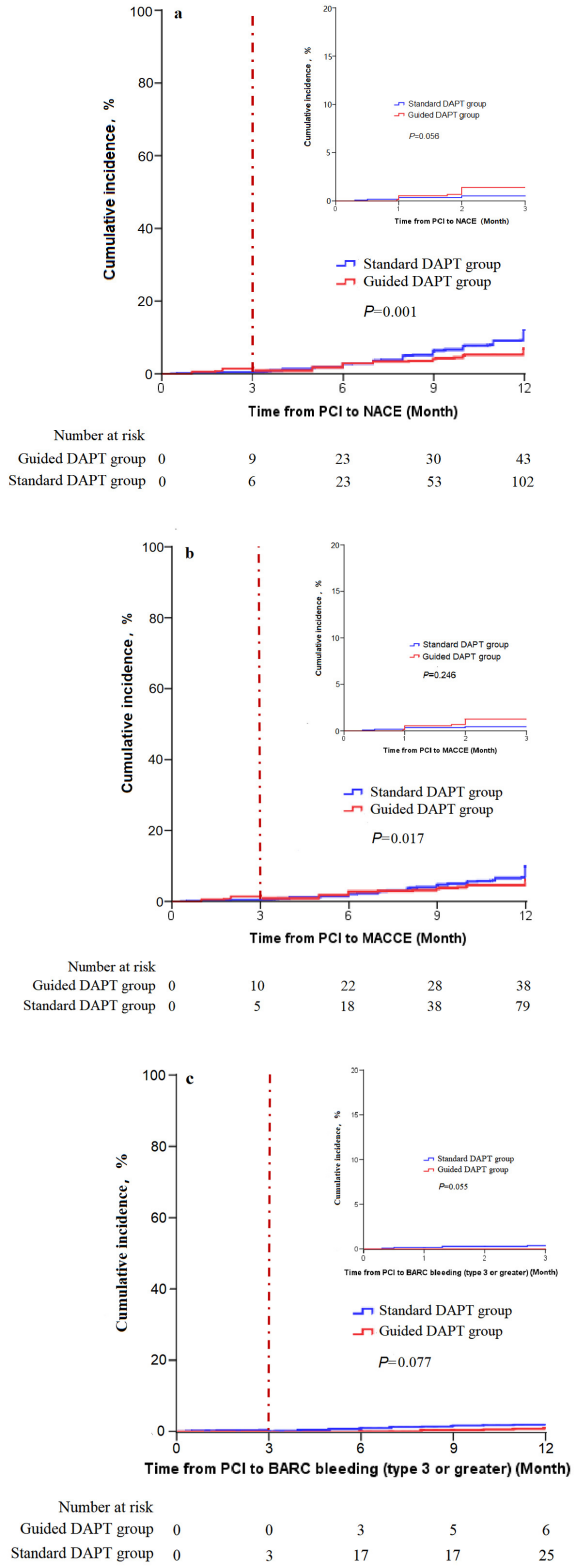
**Cumulative incidence**. (a) NACEs cumulative incidence; NACEs, net clinical adverse events, including the composite endpoint of major adverse cardiovascular and cerebrovascular events (MACCEs) and BARC bleeding (type 3 or greater). Cutoff point: 3rd month after PCI. (b) MACCEs cumulative incidence; MACCEs, major adverse cardiovascular and cerebrovascular, including the composite endpoint of cardiac death, myocardial infarction, ischemia-driven revascularization, and stroke. Cutoff point: 3rd month after PCI. (c) BARC bleeding (type 3 or greater) cumulative incidence. Cutoff point: 3rd month after PCI.

Kaplan–Meier survival curves showed that within 3 months after PCI, there were no significant differences in the incidence of NACE, MACCE, and BARC bleeding (type 3 or greater) between the two groups (all *p* > 0.05). Between 3 and 12 months after PCI, the incidence of NACE and MACCE in the guided DAPT group was significantly lower than that in the standard DAPT group (*p* = 0.001 and *p* = 0.017, respectively). Between 3 and 12 months after PCI, there was no statistically significant difference in the incidence of BARC bleeding (type 3 or greater) between the two groups (*p* = 0.077) (Fig. 2).

### 3.7 Multivariate Cox Regression Analysis

Regarding NACE and MACCE within 3–12 months after PCI, variables with statistically significant differences in baseline characteristics (presentation, primary PCI, total cholesterol, low-density lipoprotein, high-density lipoprotein, ostial lesions, diffuse lesions, small vessel disease, complete revascularization, and number of stents) as well as variables that may significantly impact the outcome measures (age, male, BMI, hypertension, type 2 diabetes, cerebrovascular disease, history of myocardial infarction, smoking history, and proximal left anterior descending artery lesions) were included as independent variables in the Cox regression analysis. The results showed that the risks of NACE and MACCE in the guided DAPT group were 0.543 times (hazard ratio [HR]: 0.543, 95% confidence interval [CI]: 0.363–0.812, *p* = 0.003) and 0.570 times (HR: 0.570, 95% CI: 0.364–0.893, *p* = 0.014), respectively, compared to the standard DAPT group. The risk of NACE and MACCE in patients with a history of cerebrovascular disease was 1.544 times (HR: 1.544, 95% CI: 1.025–2.327, *p* = 0.038) and 1.821 times (HR: 1.821, 95% CI: 1.155–2.870, *p* = 0.010) higher than that in patients without such a history. For each additional stent implanted, the risk of MACCE increased to 1.345 times the original risk (HR: 1.345, 95% CI: 1.013–1.786, *p* = 0.040) (Table 6).

**Table 6.** **Cox regression**.

Endpoints	Variables	Quotient		*Wald*	*HR*	*95% CI*	*p*
		*B*	*SE*				
NACE	Guided DAPT group	–0.611	0.206	8.835	0.543	0.363–0.812	0.003
	Male	0.357	0.198	3.273	1.430	0.971–2.105	0.070
	Age	0.017	0.010	2.778	1.018	0.997–1.039	0.096
	BMI	0.035	0.033	1.154	1.036	0.972–1.104	0.283
	UAP	0.138	0.236	0.339	1.148	0.722–1.825	0.560
	Primary PCI	0.217	0.260	0.699	1.243	0.746–2.070	0.403
	Hypertension	0.097	0.209	0.214	1.101	0.731–1.658	0.644
	Type 2 diabetes	–0.201	0.201	1.001	0.818	0.551–1.213	0.317
	Cerebrovascular disease	0.435	0.209	4.323	1.544	1.025–2.327	0.038
	OMI	–0.356	0.422	0.713	0.700	0.306–1.602	0.399
	Current smoker	0.224	0.185	1.466	1.251	0.871–1.799	0.226
	TC	–0.102	0.138	0.554	0.903	0.689–1.182	0.457
	LDL-C	0.208	0.181	1.329	1.232	0.864–1.756	0.249
	HDL-C	0.039	0.364	0.011	1.040	0.509–2.124	0.915
	Ostial lesion	–0.350	0.212	2.709	0.705	0.465–1.069	0.100
	Diffused lesion	–0.142	0.180	0.621	0.868	0.610–1.235	0.431
	Small vessel	0.075	0.263	0.082	1.078	0.644–1.805	0.775
	Complete RV	–0.209	0.216	0.940	0.811	0.531–1.238	0.332
	LADp	0.178	0.219	0.662	1.195	0.778–1.833	0.416
	Number of stents	0.204	0.132	2.376	1.226	0.946–1.589	0.123
MACCE	Guided DAPT group	–0.563	0.229	6.034	0.570	0.364–0.893	0.014
	Male	0.367	0.226	2.627	1.443	0.926–2.250	0.105
	Age	0.012	0.012	0.992	1.012	0.989–1.036	0.319
	BMI	0.039	0.036	1.125	1.039	0.968–1.116	0.289
	UAP	–0.123	0.264	0.218	0.884	0.527–1.484	0.641
	Primary PCI	0.016	0.297	0.003	1.016	0.567–1.820	0.958
	Hypertension	–0.049	0.232	0.045	0.952	0.604–1.499	0.831
	Type 2 diabetes	–0.295	0.233	1.610	0.744	0.472–1.175	0.205
	Cerebrovascular disease	0.599	0.232	6.669	1.821	1.155–2.870	0.010
	OMI	–0.090	0.427	0.044	0.914	0.396–2.111	0.834
	Current smoker	0.221	0.211	1.090	1.247	0.824–1.886	0.296
	TC	–0.146	0.154	0.906	0.864	0.639–1.167	0.341
	LDL-C	0.247	0.201	1.513	1.280	0.864–1.898	0.219
	HDL-C	0.297	0.389	0.581	1.346	0.627–2.887	0.446
	Ostial lesion	–0.389	0.243	2.557	0.678	0.421–1.092	0.110
	Diffused lesion	–0.159	0.205	0.603	0.853	0.570–1.275	0.438
	Small vessel	0.005	0.313	0.000	1.005	0.545–1.854	0.987
	Complete RV	–0.133	0.246	0.293	0.875	0.540–1.418	0.588
	LADp	0.204	0.244	0.697	1.226	0.760–1.978	0.404
	Number of stents	0.296	0.145	4.202	1.345	1.013–1.786	0.040
Cardiac death	Guided DAPT group	–1.389	0.635	4.784	0.249	0.072–0.866	0.029
	Male	0.581	0.492	1.397	1.788	0.682–4.687	0.237
	Age	0.052	0.029	3.136	1.053	0.994–1.116	0.077
	Primary PCI	–0.594	0.569	1.091	0.552	0.181–1.684	0.296
	Type 2 diabetes	–0.511	0.569	0.805	0.600	0.197–1.830	0.370
	Cerebrovascular disease	0.704	0.494	2.031	2.021	0.768–5.321	0.154
	Complete RV	0.218	0.539	0.163	1.243	0.432–3.575	0.686
	Number of stents	0.547	0.275	3.963	1.728	1.009–2.960	0.047
Stroke	Guided DAPT group	–1.751	0.541	10.483	0.174	0.060–0.501	0.001
	Male	0.377	0.388	0.948	1.459	0.682–3.118	0.330
	Age	–0.024	0.021	1.350	0.976	0.938–1.017	0.245
	Primary PCI	–0.325	0.443	0.536	0.723	0.303–1.724	0.464
	Type 2 diabetes	–0.760	0.439	2.996	0.468	0.198–1.106	0.083
	Cerebrovascular disease	1.498	0.364	16.982	4.473	2.194–9.120	<0.001
	Complete RV	–0.900	0.527	2.918	0.407	0.145–1.142	0.088
	Number of stents	0.521	0.231	5.079	1.683	1.070–2.647	0.024
BARC bleeding (type 3 or greater)	Guided DAPT group	–0.620	0.476	1.695	0.538	0.212–1.368	0.193
	Male	0.213	0.407	0.273	1.237	0.557–2.748	0.602
	Age	0.047	0.023	4.012	1.048	1.001–1.097	0.045
	Primary PCI	0.973	0.451	4.642	2.645	1.092–6.406	0.031
	Type 2 diabetes	0.418	0.413	1.022	1.519	0.675–3.415	0.312
	Cerebrovascular disease	–0.194	0.546	0.126	0.824	0.283–2.403	0.723
	Complete RV	–0.480	0.461	1.083	0.619	0.250–1.529	0.298
	Number of stents	–0.065	0.347	0.035	0.938	0.475–1.851	0.853

RV, revascularization; LADp, proximal segment of left anterior descending.

Regarding cardiac death and stroke within 3–12 months after PCI, variables that may significantly impact the outcome measures (age, male, primary PCI, type 2 diabetes, cerebrovascular disease, complete revascularization, and number of stents) were included as independent variables in the Cox regression analysis. The results showed that the risks of cardiac death and stroke in the guided DAPT group were 0.249 times (HR: 0.249, 95% CI: 0.072–0.866, *p* = 0.029) and 0.174 times (HR: 0.174, 95% CI: 0.060–0.501, *p* = 0.001), respectively, compared to the standard DAPT group. For each additional stent implanted, the risk of cardiac death increased to 1.728 times the original risk (HR: 1.728, 95% CI: 1.009–2.960, *p* = 0.047). The risk of stroke in patients with a history of cerebrovascular disease was 4.473 times higher than that in patients without such a history (HR: 4.473, 95% CI: 2.194–9.120, *p* < 0.001). For each additional stent implanted, the risk of stroke increased to 1.683 times the original risk (HR: 1.683, 95% CI: 1.070–2.647, *p* = 0.024) (Table 6).

Regarding BARC bleeding (type 3 or greater) within 3–12 months after PCI, variables that may significantly impact the outcome measures (age, male, primary PCI, type 2 diabetes, cerebrovascular disease, complete revascularization, and number of stents) were included as independent variables in the Cox regression analysis. For each additional age, the risk of BARC bleeding (type 3 or greater) increased to 1.048 times the original risk (HR: 1.048, 95% CI: 1.001–1.097, *p* = 0.045). The risk of BARC bleeding (type 3 or greater) in patients undergoing primary PCI was 2.645 times higher than that in patients not undergoing primary PCI (HR: 2.645, 95% CI: 1.092–6.406, *p* = 0.031) (Table 6).

### 3.8 Subgroup Analysis and Sensitivity Analysis

To verify the robustness of the results, this study conducted a subgroup analysis based on gender, age, presentation, primary PCI, cerebrovascular disease, current smoker, OPT-CAD, ARC-HBR, ostial lesion, diffused lesion, small vessel, complete revascularization, number of stents, and post-October 2020 era. In the subgroups of >75, STEMI, primary PCI, low risk (OPT-CAD), high risk (OPT-CAD), diffused lesion, small vessel, complete revascularization and single stent, there was no statistically significant difference in the incidence of NACE between the two groups (all *p* > 0.05). The analysis results of other subgroups are consistent with the overall results (all *p* ≤ 0.05).

**Fig. 3.**
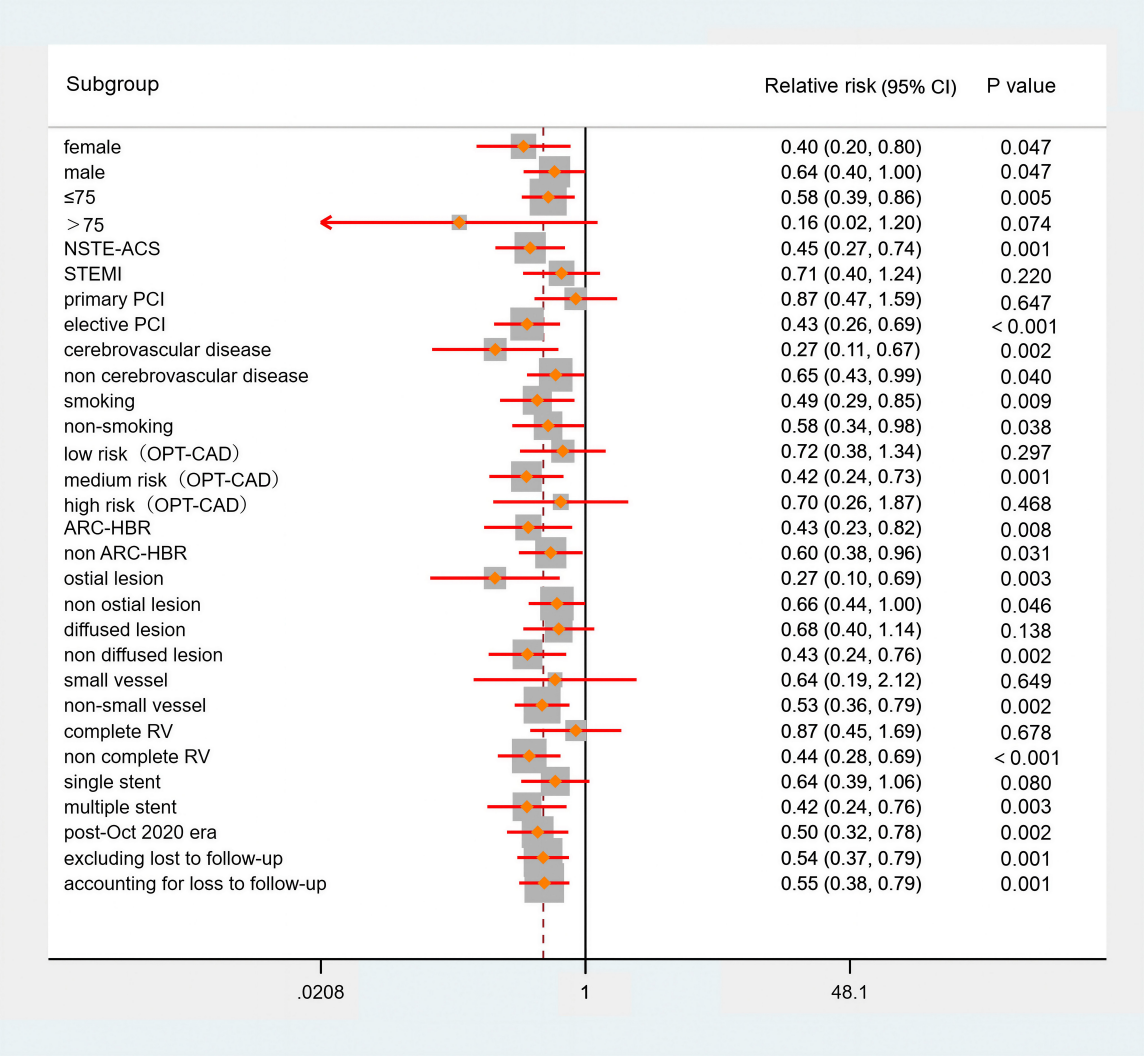
**Sensitivity analysis (NACE)**. NSTE-ACS, non-ST-segment elevation acute coronary syndrome; OPT-CAD, optimal antiplatelet antiplatelet therapy for Chinese patients with coronary artery disease.

This study included a certain proportion of patients lost to follow-up, with two cases in the guided DAPT group and four cases in the standard DAPT group. A sensitivity analysis was conducted to assess the potential impact of loss to follow-up on the results. The NACE analysis was repeated on two hypothetical datasets: one assuming that all patients lost to follow-up experienced endpoints and the other assuming that none of the patients lost to follow-up experienced endpoints. The results from both hypothetical datasets revealed that the incidence of NACE in the guided DAPT group was significantly lower than that in the standard DAPT group (*p* = 0.001 and *p* = 0.001), indicating that the study results are relatively robust (Fig. 3).

## 4. Discussion

This was a real-world, single-centre cohort study that retrospectively analysed 1816 ACS patients at a dual high risk who underwent PCI. The conclusions are as follows. (1) A significant proportion of ACS patients undergoing PCI exhibit the dual high-risk features, accounting for approximately 55.09% (1822/3307). (2) The PFT-guided DAPT scheme selection is recommended for patients with dual high-risk ACS who have undergone PCI. Compared to DAPT regimens subjectively chosen by clinicians based on experience, this approach can significantly benefit from NACE (a composite endpoint of MACCE and BARC bleeding (type 3 or greater)) and MACCE.

For ACS patients undergoing PCI, current guidelines recommend using a 12-month combination of aspirin and potent P2Y_12_ receptor inhibitors for DAPT. Although intensified or prolonged DAPT treatment can reduce the risk of ischemia, it can increase bleeding events, which is a predictive factor for poor prognosis. The risk of ischemia in patients with ACS is time-dependent and gradually decreases over time. Therefore, in clinical practice, efforts are being made to balance bleeding and ischemia risks through unguided de-escalation strategies, in order to achieve personalized treatment [10]. For patients at a dual high risk for ischemia and bleeding, there is an even greater need to balance ischemic and bleeding risks. In this study, the prevalence of dual high-risk ACS patients was approximately 60%, notably higher than the 32% reported using the “evaluating the performance of the can rapid risk stratification of unstable angina patients suppress adverse outcomes with early implementation of the ACC/AHA guidelines (CRUSADE)”-“global registry of acute coronary events (GRACE)” risk combinations [11]. This result differs significantly from our findings. A likely explanation for this discrepancy is the divergent methodological approaches used to define dual high-risk status. Additionally, while the aforementioned study focused on ACS patients, our study exclusively included ACS patients who underwent PCI. This suggests that the coronary artery disease severity in our cohort may be greater, potentially explaining the higher proportion of dual high-risk patients. Similarly, a study applying the OPT-BIRISK criteria reported a comparable dual high-risk prevalence of 58.8% (4146/7049) in ACS patients who underwent PCI, which is closely aligned with our findings [12]. However, current evidence offers little guidance on DAPT regimens for dual high-risk patients. In 2020, the ESC guidelines proposed a preliminary antiplatelet drug transition model based on ischemic and bleeding risk. Bleeding risk was assessed in NSTE-ACS patients who underwent PCI. For high bleeding risk patients, de-escalation to aspirin monotherapy is recommended at the 3-month post-PCI. For remarkably high bleeding risk patients, de-escalation to clopidogrel monotherapy is suggested at the 1-month post-PCI. For low bleeding risk patients, further assessment of high ischemic risk is required to develop a more precise antiplatelet strategy [3]. However, this model lacks discussion on antiplatelet regimens for dual high-risk patients, highlighting the gap in evidence in the guidelines. For dual high-risk patients, an additional tool beyond the current risk scoring systems is required to assist clinicians in selecting DAPT regimens. However, ischemic and bleeding events in ACS patients undergoing PCI result from the synergistic effects of multiple factors, including age and comorbidities such as diabetes, hypertension, atrial fibrillation, heart failure, complex CAD, procedural factors, and anticoagulants or nonsteroidal anti-inflammatory drugs [3, 5, 13, 14, 15]. In recent years, studies on PFT-guided DAPT for patients with ACS have not resulted in definitive guidelines [3, 8, 9]. PFT can individually reflect platelet activation and aggregation capacity, ensuring adequate platelet inhibition during the long-term treatment of patients with ACS. Previous studies identified high platelet reactivity as an independent risk factor for MACCE within 12 months after PCI. Conversely, it was negatively correlated with bleeding events [16, 17]. In the ANTARCTIC study, adjustments were made for DAPT escalation or de-escalation based on PFT [8, 18]. Compared to medication adjustments based on experience, PFT-guided medication adjustments can reduce the risks of cardiac death, myocardial infarction, in-stent thrombosis, and stroke, as well as minor bleeding risks [9]. However, other studies suggested that PFT-guided DAPT does not reduce the occurrence of ischemic and bleeding events [19, 20]. A potential explanation is that PFT alone has a limited role in guiding DAPT. In contrast, risk-scoring systems for patients with ACS encompass various high-risk factors beyond PFT. Therefore, it is important to determine how PFT can be combined with risk scores to guide the selection of DAPT regimens selection. In other diseases, combining scoring systems with biomarkers to guide diagnosis and treatment is common, such as combining the results of the Wells and Geneva scores with D-dimer levels to assist in the identification of pulmonary embolism [21].

A study has shown that the association between low platelet reactivity and bleeding events is weaker than the association between high platelet reactivity and thrombotic complications [22]. This may be because the optimal cutoff values for platelet reactivity related to high bleeding and ischemic risks are not yet clearly defined [23, 24]. However, because the ischemic risk in ACS patients gradually decreases after PCI while the bleeding risk increases. Consequently, the bleeding risk surpasses the ischemic risk at one month post-ACS onset, after which both risks tend to stabilize [25]. Since bleeding and ischemic risks vary at different stages following PCI, the optimal therapeutic window for platelet reactivity may differ. The most convenient approach is to perform PFT to guide the DAPT scheme selection when a patient’s ischemic and bleeding risks begin to stabilize, which is also the optimal time for medication transition. In this study, PFT was conducted at 3 months after PCI to guide the DAPT strategy selection. Most patients in this study underwent outpatient follow-up at 3 months after PCI; however, evidence confirms that potent P2Y_12_ inhibitors maintain a critical advantage in preventing early-phase ischemic events within the first month of ACS [26]. This suggests that although the bleeding risk begins to exceed the ischemic risk at 1 month after PCI, the ischemic risk remains relatively high, and transitioning medications at this stage may increase the occurrence of ischemic events, particularly when de-escalating antiplatelet drugs are used. Despite the continuing increase in the risk of in-stent thrombosis within six months post-PCI with newer-generation drug-eluting stents, in-stent thrombosis has been shown not to significantly differ between 3 and 6 months after PCI [27]. Therefore, in our study, we chose to adjust antiplatelet therapy and perform PFT at 3 months after PCI. In addition, because the distribution of cytochrome P450 family 2 subfamily C member 19 (CYP2C19) alleles varies among African, European, and East Asian populations and these alleles can influence platelet reactivity [28]. There is a need to identify the optimal window for platelet reactivity across ethnic groups, especially considering the ‘East Asian paradox’. Owing to the inclusion of East Asian populations in this study, there may be certain limitations in the generalizability of the results.

In real-world practice, the choice of P2Y_12_ receptor inhibitors for post-PCI patients typically considers three aspects: the assessment results of the ischemia and bleeding risks, the complexity of lesions during PCI, and whether the patient has respiratory distress and gout caused by ticagrelor. Given that the population included in this study were all at double high risk, and the complexity of lesions was also included in the assessment of ischemic risk, the use of ticagrelor in this study was based on the subjective habits and experience of different clinical doctors. An analysis was performed to assess the potential confounding effect of ticagrelor use at discharge, which revealed no significant difference between the two groups (*p* = 0.200). De-escalation strategies for DAPT include switching to a less potent P2Y_12_ receptor inhibitor, dosage reduction, and shortening the duration of DAPT. Clearly, De-escalation ticagrelor from the standard dose (90 mg BID) to a lower dose (60 mg BID) represents one of these regimens. Previous pharmacodynamic and pharmacokinetic study have demonstrated that in patients with a history of myocardial infarction, the antiplatelet effect of low-dose ticagrelor is comparable to that of the 90 mg twice daily regimen [3]. Thus, reducing ticagrelor from the standard dose to the lower dose. Its essence is a “response-guided de-escalation”. Rationale for reducing platelet related from 90 mg to 60 mg is a continuation of strong platelet inhibition. And it is applicable to patients who still have significant ischemic risk (such as complex lesions, diabetes, previous myocardial infarction), but cannot tolerate the risk of bleeding at a dose of 90 mg. The strategy of switching from ticagrelor 90 mg to clopidogrel 75 mg is primarily suitable for patients with bleeding risk outweighing ischemic risk, those intolerant to ticagrelor, and those who prefer or require clopidogrel due to personal preference or economic considerations.

The results of this study indicate that PFT-guided DAPT strategy selection can benefit patients in terms of NACE, MACCE, cardiac death, and stroke without increasing the risk of BARC bleeding (type 3 or greater). The results in this dual high-risk ACS population are corroborated by other studies of PFT-guided DAPT [20, 29]. The “Guided de-escalation of antiplatelet treatment in patients with acute coronary syndrome undergoing percutaneous coronary intervention (TROPICAL-ACS)” study analysis showed that for acute myocardial infarction patients post-PCI, after 7 days of prasugrel and clopidogrel treatment, those with low platelet reactivity,who de-escalated to clopidogrel had a lower incidence of the primary endpoints (cardiac death, myocardial infarction, stroke, and BARC ≥2 bleeding) at 1 year compared to those who continued prasugrel (7.3% vs. 9.0%, *p* < 0.001, HR: 0.81, 95% CI: 0.62–1.06), with increased clinical benefit and no inferiority in ischemic events such as in-stent restenosis, all-cause death, and urgent revascularization [20]. A pre-specified analysis showed that guided de-escalation to clopidogrel based on low platelet reactivity at one month post-PCI reduced both ischemic and bleeding events at one year, resulting in a net clinical benefit [29]. In contrast to the timing of platelet inhibition after acute coronary syndrome (TOPIC) trial, our analysis failed to identify guided DAPT as an independent predictor of major bleeding events. The limited sample size resulted in a low number of BARC bleeding (type 3 or greater) events and a potential reduction in statistical power. However, the PFT method used in this study was LTA, which is considered the “gold standard” [22], but lacks a clear cutoff value for low platelet reactivity and is influenced by thrombocytopenia and thrombocytosis. Although this study excluded patients with thrombocytopenia and thrombocytosis, the cutoff value for low platelet reactivity in this study was set at MAR(ADP) ≤30%, which may require further study.

Although the prognostic impact of guided DAPT in dual high-risk ACS patients has not been established, similar study have been conducted [8]. The ANTARCTIC study was a randomized controlled trial (RCT) conducted across 35 centres in France. This trial enrolled 877 elderly (age ≥75) patients with ACS after stent implantation and randomly assigned them to either a monitored group or a conventional group. In the monitored group, adjustments were made based on the results of VerifyNow. The conventional group received a fixed dose of prasugrel (5 mg/day) without any adjustments or PFT. No significant differences were observed between the two groups in the MACE within 12 months post-PCI [8]. The study population comprised elderly patients, and according to the OPT-BIRISK trial definition of dual high-risk patients, any patient aged ≥75 years was considered to have a dual high risk without further assessment of other risk factors for ischemia or bleeding. Relying solely on age to determine whether a patient carries a dual high risk is inaccurate. Therefore, although the population in this study was similar to that in the ANTARCTIC study, there were certain differences that may explain the inconsistent conclusions between the two studies. The relevant consensus suggests that when clinical judgment indicates a balance between ischemic and bleeding risks, PFT-guided DAPT can demonstrate certain advantages [22]. This balance typically includes two scenarios—namely, low ischemic risk with low bleeding risk and high ischemic risk with high bleeding risk. The latter scenario has a lower tolerance to inappropriate DAPT regimens than the former, necessitating individualised treatment. This innovative aspect of this study addresses clinical challenges in real-world practice. The results of this study show that the main difference between antiplatelet agents across the groups is represented by the number of patients on ticagrelor 60 mg (5.9% vs. 2.3%), which may explain the lower incidence of major bleeding and stroke according to the “prevention of cardiovascular events in patients with prior heart attack using ticagrelor compared to placebo on a background of aspirin (PEGASUS)” trial [30]. Therefore, we would stress that, patients with dual high-risk may more likely benefit from such approach rather than standard DAPT regimens, as supported by previous pharmacodynamic data [31]. On the other hand, due to the low number of plaques on ticagrelor 60 mg in this study, and the fact that 218 (30.6%) patients completed the conversion at 3 months after PCI, the guided selection of antiplatelet regulation is more likely to have been the one ultimate leading to the results.

Cox regression analysis revealed that in addition to guided DAPT being a protective factor for NACE, MACCE, cardiac death, and stroke, cerebrovascular disease, age, and number of stents were identified as risk factors for ischemic events. Primary PCI is a risk factor for bleeding. These findings are consistent with those of multiple studies and ischemic or bleeding risk scoring systems [4, 5, 7, 32, 33, 34, 35]. These factors directly increase the risk of NACE. The ACS guidelines, developed by the ESC in 2023, propose criteria for high ischemic risk, including complex CAD [32]. The OPT-CAD, patterns of non-adherence to antiplatelet regimens in stented patients-coronary artery thrombosis event (PARIS-CTE), and DAPT scoring systems also include multiple risk factors, such as smoking [4, 5]. This study adopted the risk evaluation system from the OPT-BIRISK trial, which is more suitable for the Chinese ACS population. Compared with other scoring systems, it incorporates PCI-related factors. This approach provides a comprehensive risk profile by simultaneously evaluating both ischemic and bleeding in the ACS patients undergoing PCI [7]. Since the population of the OPT-BIRISK trial was Chinese, it was more appropriate to evaluate the Chinese population in this study. The risk assessments in this study were conducted during patient hospitalization, and no dynamic assessment was implemented at 3 months after PCI. However, the OPT-BIRISK trial included indicators such as GFR and creatinine level, which can be improved with standardized treatment. Improvements in these indicators might lead to changes in the dual high-risk assessment results for ischemia and bleeding at 3 months after PCI. In this study, the decision not to perform dynamic assessments was primarily based on the low proportion of patients with eGFR <60 mL/min/1.73 m^2^ at baseline, which had a minimal impact on the final assessment results, while the likelihood of improvement in other indicators in the scoring system was low. However, these factors can impair the response to clopidogrel. Acute clinical symptoms, diabetes, advanced age, BMI, and chronic kidney disease have been described by previous studies as clinical characteristics associated with impaired clopidogrel responsiveness [33, 34]. The “age, body mass index, chronic kidney disease, diabetes mellitus, and genotyping (ABCD-GENE)” score largely encompasses these factors and has high accuracy in predicting high platelet reactivity [35]. Despite the known influence of multiple factors on platelet reactivity, their impact on assessments of ischemic and bleeding risk remains uncertain.

## 5. Limitations

(1) This was a cohort study, and physicians subjectively determined whether to use PFT to guide the selection of DAPT protocol samples. In addition, we began conducting PFT in October 2020. The enrollment times of the patients in the two groups were not entirely synchronised, resulting in a selection bias in the study population. Moreover, owing to the limitation of not conducting random grouping in the cohort study, there were certain confounding factors in this research. To address potential confounding, baseline characteristics were thoroughly analyzed, and a Cox regression model was employed to control for their influence on the outcomes. (2) Currently, there are advantages and disadvantages to the assessment systems for the risk of ischemia and bleeding. This study adopted the definition of dual high risk established in the OPT-BIRISK trial. Although it is the most suitable risk assessment system for this study, it has the inherent limitation of not comprehensively assessing risks. The OPT-BIRISK criteria are trial-specific and their generalisability is questionable. (3) The current analysis was performed on East-Asian patients, a population with distinct ischemic and bleeding risk profiles compared to other ethnic groups. (4) LTA was used in this study for PFT. However, the cutoff values for high and low platelet reactivity obtained using different PFT methods are inconsistent, and the conversion of platelet reactivity between different testing methods is currently unclear. Therefore, further research is needed to explore whether other PFT methods could benefit ACS patients at a dual high risk for ischaemia and bleeding. (5) Since this was a real-world study, patients in the control group received various DAPT regimens. Future studies should compare PFT-guided DAPT regimens with de-escalation, escalation, extension, and shortening of DAPT to provide more evidence for PFT-guided DAPT and to refine antiplatelet therapy strategies. (6) At present, there is no recognized clinical cut-off value for the treatment of platelet dysfunction worldwide. So each laboratory should develop a cutoff value related to clinical outcomes based on their own treatment (medication) situation, testing methods, and clinical practice. The normal range of ADP for platelet function testing in our center is 52–84%, and the safe range for ADP treatment is 30–50%. At the same time, drawing on relevant research [22, 36], we have set the following standards: When the MAR(ADP) >50% cut-off, escalation should be considered. When The MAR(ADP) ≤30% cut-off, de-escalation should be considered.

## 6. Conclusion

The primary recommendation for patients with ACS with dual high risk among the East-Asian population is to use PFT to guide DAPT within 12 months after PCI, as it can reduce the incidence of NACE, primarily by lowering MACCE. However, the conclusions of this study need to be further verified in RCTs with a larger sample size.

## Availability of Data and Materials

All data generated or analyzed during this study are included in this published article [and its supplementary information files].

## Supplementary Material

Supplementary material associated with this article can be found, in the online version, at https://doi.org/10.31083/RCM41544.
