# Supplementary figures and images for: The Impact of Dual Antiplatelet Therapy Guided by Platelet Function Testing on the Prognosis of Patients With Dual High-Risk Acute Coronary Syndrome Undergoing Percutaneous Coronary Intervention
Source: Rev Cardiovasc Med. 2026 Feb 11;27(2):41544. doi: 10.31083/RCM41544 (PMC12960008; doi:10.31083/RCM41544)

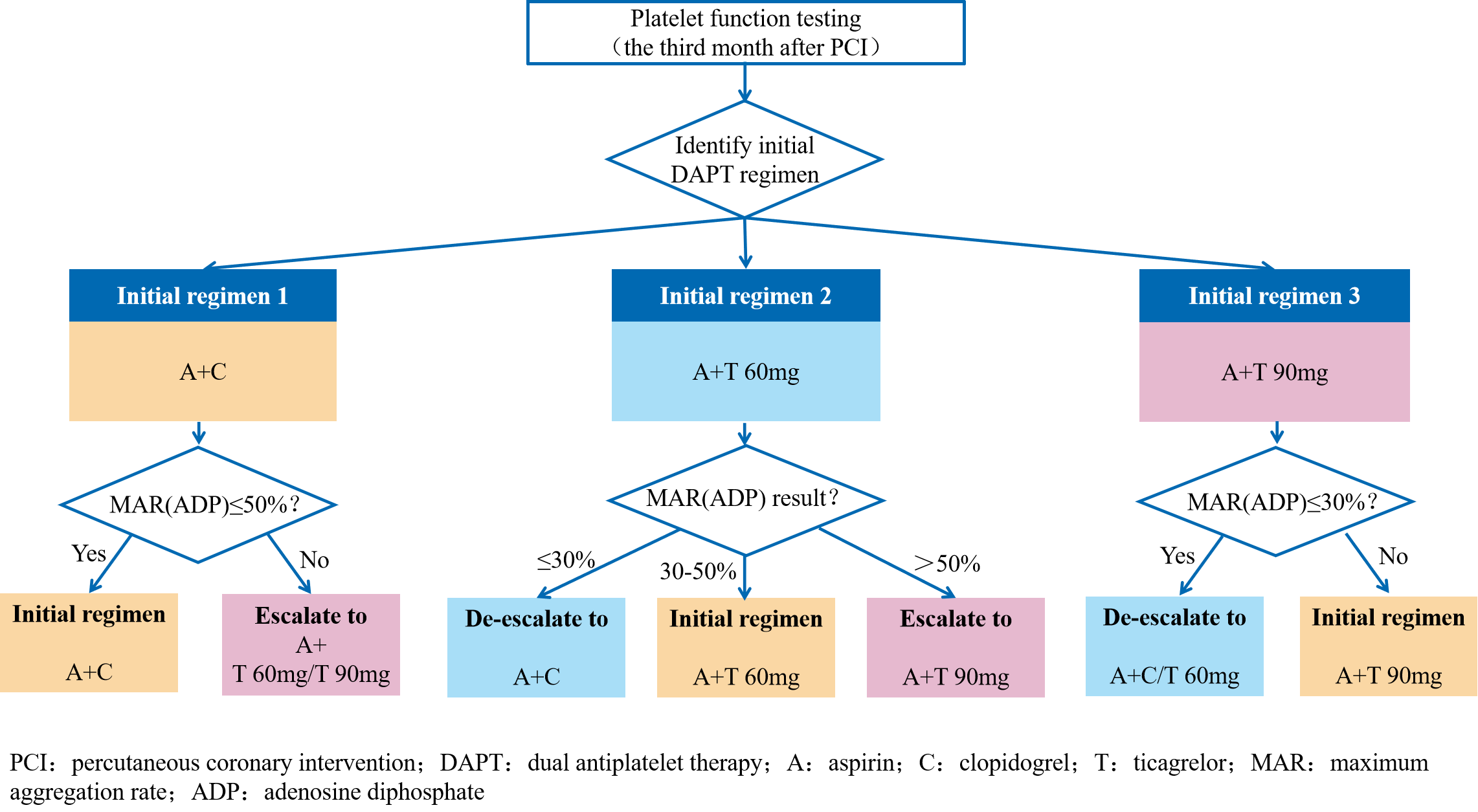

Supplement: Supplementary file 1 [file 2153-8174-27-2-41544-s1.zip › Supplementary material 2.tif]
