# Supplementary material for: The Impact of Dual Antiplatelet Therapy Guided by Platelet Function Testing on the Prognosis of Patients With Dual High-Risk Acute Coronary Syndrome Undergoing Percutaneous Coronary Intervention
Source: Rev Cardiovasc Med. 2026 Feb 11;27(2):41544. doi: 10.31083/RCM41544 (PMC12960008; doi:10.31083/RCM41544)
Supplement: Supplementary file 1 [file 2153-8174-27-2-41544-s1.zip › Supplementary Material.doc]

### **Supplementary Material 1**

### **a.Detailed Platelet Function Testing (LTA) Protocol**

**Agonist**: Light transmission aggregometry (LTA) was performed using adenosine diphosphate (ADP; Sigma-Aldrich) at a final stimulating concentration of 5 μM.

**Calibration & Quality Control:** The aggregometer was calibrated daily using platelet-poor plasma as 100% light transmission and autologous platelet-rich plasma (PRP) as 0% aggregation. Commercial control plasmas with normal and abnormal aggregation responses (HYPHEN BioMed) were run with each batch to ensure assay performance.

**Timing of Blood Sampling:** In this study, patients in the guided DAPT group had their first blood sampling collected 3 months after PCI. For patients who needed to adjust the type or dosage of **P2Y₁₂ inhibitor**, blood sampling was collected and tested again one week after regular medication.

**Pre-analytic Handling:** The fasting venous blood was obtained from the elbow at 3 months after PCI. 3 ml of venous blood was collected into a coagulation vacuum tube, thoroughly mixed for anticoagulation, and tested within 3 hours at room temperature. PRP was obtained by centrifugation at 150 × g for 10 minutes; platelet-poor plasma (PPP) was obtained by further centrifugation at 2000 × g for 10 minutes.

**Assay Variability**: Intra-assay and inter-assay coefficients of variation (CV) for our LTA-ADP (20 μM) were 4.8% and 8.2%, respectively.

### **b. Rationale for MAR(ADP) Cut-off Values**

We have added the following justification and references for the selected cut-offs:

At present, there is no recognized clinical cut-off value for the treatment of platelet dysfunction worldwide. So each laboratory should develop a cutoff value related to clinical outcomes based on their own treatment (medication) situation, testing methods, and clinical practice. The normal range of ADP for platelet function testing in our center is 52-84%, and the safe range for ADP treatment is 30-50%. At the same time, drawing on relevant research（Breet NJ, van Werkum JW, Bouman HJ, Kelder JC, Ruven HJ, Bal ET, et al. Comparison of platelet function tests in predicting clinical outcome in patients undergoing coronary stent implantation. JAMA. 2010 Feb 24;303(8):754-62. doi: 10.1001/jama.2010.181. Angiolillo DJ, Galli M, Alexopoulos D, Aradi D, Bhatt DL, Bonello L, et al. International Consensus Statement on Platelet Function and Genetic Testing in Percutaneous Coronary Intervention: 2024 Update. JACC Cardiovasc Interv. 2024 Nov 25;17(22):2639-2663. doi: 10.1016/j.jcin.2024.08.027.）, we have set the following standards: When The MAR (ADP)>50% cut-off, escalation should be considered. When The MAR (ADP) ≤ 30% cut-off, de-escalation should be considered.

We acknowledge that these thresholds are not universally standardized and their clinical utility is still under investigation. Our aim was to adopt a response-guided strategy based on the existing literature and our institutional experience.

### **c. Concordance with Other Platforms & Local Validation：the comparison between LTA and other platforms is listed in the following table**

| Test | Principle | Advantages | Drawbacks |
| --- | --- | --- | --- |
| LTA | Platelet aggregation | Historical gold standard | (1) Large sample volume (2) Influenced by low platelet count (3) Complex sample preparation (uses PRP and PPP) (4) Requiring expert personnel (5) Centrifugation may affect platelet reactivity (6) Time-consuming |
| VASP phosphorylation | Flow cytometry | (1) Whole-blood assay (2) Very small sample volume (3) Specifically assesses the P2Y_12_ signaling pathway (4) Can be shipped to core lab | (1) Complex sample preparation  (2) Requires flow cytometer and experienced technician Expensive  (3) Insensitive to low levels of P2Y12 blockade |
| VerifyNow | Platelet aggregation | (1) Whole-blood assay (2) Small sample volume (3) Very simple and rapid (4) No sample preparation (5) True point of care(no pipetting required) (6) Most extensive evidence for prognostic value | (1) Influenced by hematocrit and platelet count range (2) Expensive |
| Multiplate Analyzer | Multiple electrode aggregometry | (1) Whole-blood assay (2) Small sample volume Simple and rapid | (1) Influenced by low platelet count (2) Requires sample dilution |
| TEG Platelet Mapping system | Platelet contribution to clot strength | (1) Whole-blood assay (2) Global evaluation of hemostasis (clot formation and lysis) | (1) Limited studies (2) Requires experienced technician |

### **Supplementary Material 2**
